# Guest-Mediated Reversal of the Tumbling Process in Phosphorus-Dendritic Compounds Containing β-Cyclodextrin Units: An NMR Study

**DOI:** 10.3390/ph14060556

**Published:** 2021-06-11

**Authors:** Kendra Sorroza-Martínez, Israel González-Méndez, Mireille Vonlanthen, Fabián Cuétara-Guadarrama, Javier Illescas, Xiao Xia Zhu, Ernesto Rivera

**Affiliations:** 1 Instituto de Investigaciones en Materiales, Universidad Nacional Autónoma de México, Circuito Exterior, Ciudad Universitaria, México City CP 04510, Mexico; kendraivn17@gmail.com (K.S.-M.); mireille.vonlanthen@gmail.com (M.V.); fabian.cuetara@comunidad.unam.mx (F.C.-G.); 2 Tecnológico Nacional de México/Instituto Tecnológico de Toluca, Avenida Tecnológico S/N Col. Agrícola Bellavista, Metepec CP 52140, Mexico; fillescasm@toluca.tecnm.mx; 3 Département de Chimie, Université de Montréal, C.P. 6128, Succursale Centre-ville, Montreal, QC H3C 3J7, Canada; julian.zhu@umontreal.ca

**Keywords:** phosphorus dendritic compounds, β-cyclodextrin, tumbling process, host-guest interaction, 1-adamantanecarboxylic acid

## Abstract

The conformational study of dendritic platforms containing multiple β-cyclodextrin (βCD) units in the periphery is relevant to determine the availability of βCD cavities for the formation of inclusion complexes in aqueous biological systems. In this work, we performed a detailed conformational analysis in D_2_O, via 1D and 2D NMR spectroscopy of a novel class of phosphorus dendritic compounds of the type P_3_N_3_-[O-C_6_H_4_-O-(CH_2_)_n_-βCD]_6_ (where *n* = 3 or 4). We unambiguously demonstrated that a functionalized glucopyranose unit of at least one βCD unit undergoes a 360° tumbling process, resulting in a deep inclusion of the spacer that binds the cyclodextrin to the phosphorus core inside the cavity, consequently limiting the availability of the inner cavities. In addition, we confirmed through NMR titrations that this tumbling phenomenon can be reversed for all βCD host units using a high-affinity guest, namely 1-adamantanecarboxylic acid (AdCOOH). Our findings have demonstrated that it is possible to create a wide variety of multi-functional dendritic platforms.

## 1. Introduction

Dendrimers are nanometric-sized polymeric structures consisting of regularly branched layers obtained through the step-by-step incorporation of branching units (monomers) arranged around a central nucleus. Every time a new generation is created, the number of terminal functional groups is multiplied. Unlike hyperbranched polymers that are prepared via polymerization reactions, dendrimers are synthesized through consecutive and iterative steps, allowing total control of their final structure. A wide variety of dendrimers have been prepared and studied, most of which are purely organic. Furthermore, “inorganic” dendrimers [1], containing heteroatoms such as phosphorus or silicon in their molecular structure either in the nucleus or as branching points, have been reported [2,3,4]. Majoral and Caminade were pioneers in the synthesis and development of phosphorus dendrimers with a cyclotriphosphazene nucleus (hexachlorocyclotriphosphazene). These phosphorous dendrimers have found various applications [5], for instance, as anticancer agents [6], fluorescent probes, biomacromolecules, and anti-Alzheimer drugs [7]. 

The terminal groups of dendrimers determine their macroscopic properties such as solubility and reactivity; the latter allows a subsequent chemical modification of the final dendrimeric structure [8]. Surface engineering deals with the modification of the dendrimer periphery, as it employs elegant strategies to improve the physico-chemical and biological properties of dendrimers [9,10]. In recent years, the development of surface-modified dendrimers with carbohydrates, specifically with cyclodextrins (CDs), has led to a synergistic effect on the properties of the obtained constructs [11,12,13,14,15].

From the multi-component structure perspective, the structural modification of individual CD molecules is not sufficient to meet the emerging needs for developing new optimized materials with multiple applications. Therefore, the inclusion of multiple CD units into complex molecular structures has been shown to endow important features, such as excellent physico-chemical properties [16]. For this reason, in addition to dendrimer design, various CD-containing structures such as dimers [17,18,19,20,21], trimers [15,22], and tetramers [23] have been developed.

β-Cyclodextrin (βCD) is a cyclic oligosaccharide formed by seven α-D-glucopyranose units bound by α-1,4 glycosidic bonds. Its truncated cone-like shape forms a cavity with hydrophobic character resulting from the inward-directed C-H bonds. Hydrophilic properties are conferred by the -OH groups lying on the outer surface of the cone [16,24]. Even though βCD has the lowest solubility in water among the reported CDs, it is the most cited CD for use as a building block. Features of βCD such as easy and cheap production, as well as its good biocompatibility, have earned its approval by the Food and Drug Administration (FDA) to be used as an excipient. βCD is a very versatile molecule, as it can be chemically modified in its primary and secondary hydroxyl groups. If the cavity remains accessible after chemical modification, unique supramolecular structures with different functionalities can be obtained [25,26].

While studying CD-containing dimers, trimers, and tetramers, an unusual inversion phenomenon was observed in the functionalized glucopyranose unit in water. This inversion or tumbling causes the spacer that links to the CDs with the platform to deeply insert into the CD cavity. Very recently, this phenomenon has been reported on the surface of CD-functionalized PAMAM dendrimers [15]. This tumbling depends on the type of spacer used, and, when favored, has a direct negative impact on the accessibility to the cavities of the CDs present in the platform. Therefore, it is of great importance to understand and control this tumbling unit to ensure the complete availability of CDs on multi-functionalized platforms [17,18,20].

A strategy employed to reverse the tumbling process in the functionalized glucopyranose unit is the use of the self-assembly of CDs. Self-assembly is a phenomenon in which simple molecules function as building blocks to spontaneously organize into more complex systems. The process of self-organization is carried out through different interaction mechanisms: electrostatic forces, van der Waals forces, π–π interactions, dispersion forces, or more specific bonds such as the formation of host–guest complexes, or “lock -and- key” binding [27,28]. CDs stand out of all macrocyclic molecules as a gold standard for the formation of host–guest complexes [29]. Among the many molecules studied as guests of the internal cavity of βCD, special attention has been paid to adamantane (Ad) because it fits firmly into the cavity, resulting in a very stable host–guest inclusion complex [30,31]. This interaction has been studied as a strategy to reverse the phenomenon of inversion in the glucopyranose unit of the βCD [15,19]. In addition, it can be used to create a variety of functionalities on nanoparticles and surfaces containing multiple CD units [32,33,34].

In a previous work, we reported the synthesis and characterization in DMSO-*d_6_* of P_3_N_3_-[O-C_6_H_4_-O-(CH_2_)_n_-βCD]_6_ (*n* = 3 or 4) dendritic compounds [14]. However, this medium is not representative of the real conformation to assure the complete availability of βCD cavities of this type of platform in aqueous biological systems. Therefore, in this work, we presented the full characterization of P_3_N_3_-[O-C_6_H_4_-O-(CH_2_)_n_-βCD]_6_ (*n* = 3 or 4) dendritic compounds with six neighboring βCD units in deuterium oxide (D_2_O). Our aim was to elucidate the real conformation in water and identify whether the tumbling process is favored in this type of structure. Furthermore, the formation of the inclusion complex between the dendritic compounds and 1-adamantanecarboxylic acid (AdCOOH) was investigated in order to demonstrate that it is entirely possible to block the tumbling process with the Ad guest in these multifunctional platforms.

## 2. Results and Discussion

### 2.1. Characterization 

**Figure 1:**
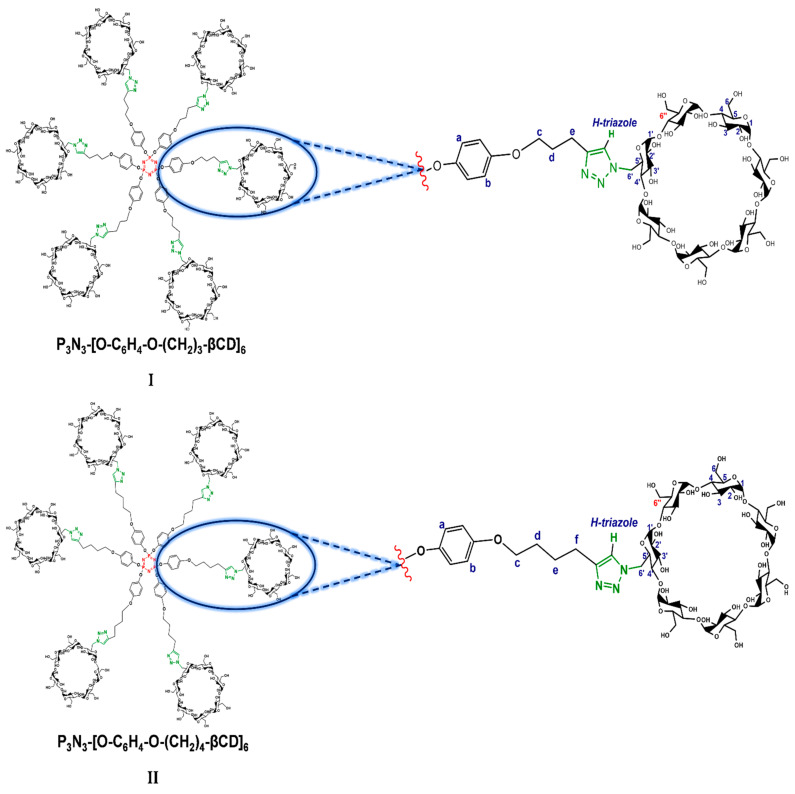
Structure of compounds P_3_N_3_-[O-C_6_H_4_-O-(CH_2_)_n_-βCD]_6_, where *n* = 3 or and 4 (**I** and **II**, respectively) and the representative assignment of the proton signals in NMR.

**Figure 2:**
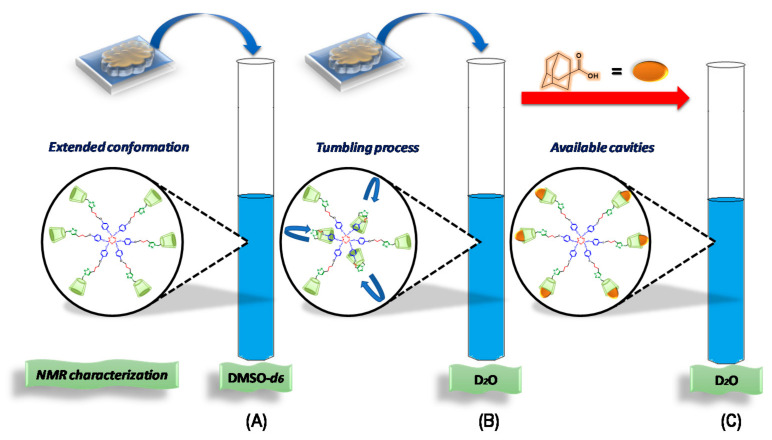
Possible scenarios for the characterization of P_3_N_3_-[O-C_6_H_4_-O-(CH_2_)_n_-βCD]_6_ (*n* = 3 or 4) compounds by NMR, (**A**) Extended conformation, (**B**) Tumbling process and (**C**) Available cavities in in dendritic compounds (**I**) and (**II**).

In a previous work, we reported the correct assignment of the ^1^H- and ^13^C-NMR signals of P_3_N_3_-[O-C_6_H_4_-O-(CH_2_)_n_-βCD]_6_ dendrimers **I** and **II**, where *n* = 3 and 4, respectively (see as (**I**) and (**II**) in Figure 1), in DMSO-*d_6_* employing HMQC and COSY 2D NMR experiments [14]. In this medium, the classical signals of the aromatic triazole ring protons and the disubstituted phenyl protons appeared as singlets, suggesting an extended conformation for the dendritic molecule (see (A) in Figure 2). This first approach to the spatial conformation of multi-functionalized P_3_N_3_-[O-C_6_H_4_-O-(CH_2_)_n_-βCD]_6_ molecules is not surprising, as it is well known that DMSO solvates each hydrogen atom of the CD OH groups, preventing the interaction with other media (for example, water) [35,36]. Besides, some DMSO molecules can penetrate inside the CD cavities, which blocks the ability of the CD to form inclusion complexes by competition of the interaction forces in the final system neighborhood [37,38].

**Figure 3:**
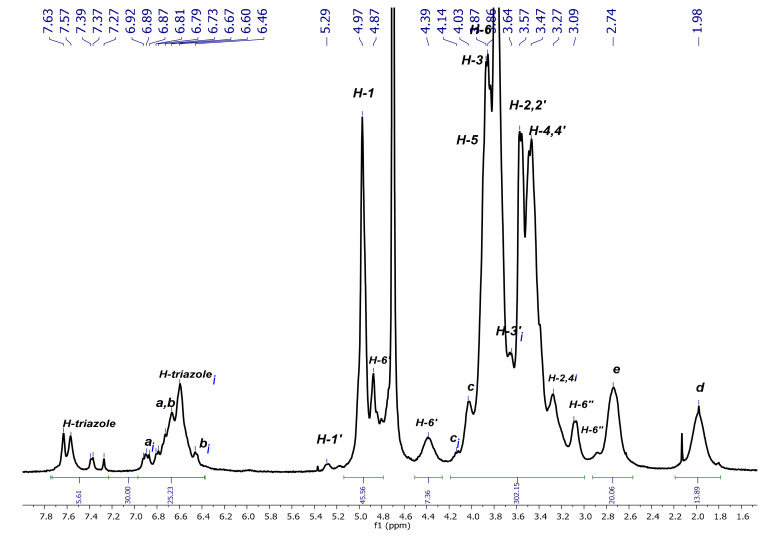
^1^H-NMR spectrum of P_3_N_3_-[O-C_6_H_4_-O-(CH_2_)_3_-βCD]_6_ (**I**) in D_2_O.

As these compounds may be potentially used as nanocarriers in biological systems, the rigorous elucidation of their conformation in aqueous media is a determinant for such applications. For this reason, we have herein pursued the assignment of the signals in D_2_O and have performed a conformational analysis via 2D NMR (through NOE interactions) of both native platforms and the corresponding inclusion complexes using AdCOOH as the guest molecule. First, the full characterization of native P_3_N_3_-[O-C_6_H_4_-O-(CH_2_)_n_-βCD]_6_ (**I**) and (**II**) using NMR techniques (^1^H-, ^13^C-NMR, and 2D NMR HMQC and COSY) (Figure 3 and Figures S1–S7 for (**II**) in SI) was carried out in D_2_O. The ^1^H-NMR spectrum of P_3_N_3_-[O-C_6_H_4_-O-(CH_2_)_3_-βCD]_6_ (**I**) showed a radical change in the aromatic region, as the signals corresponding to symmetric H-triazole and aromatic H-a, H-b protons no longer appeared as singlets, as observed for the extended conformation in DMSO (Figure 3). Instead, at least four different signals were observed between 7.63–7.27 ppm, which can be assigned to H-inverted triazoles (***i***). The same phenomenon was observed between 6.92–6.46 ppm where multiple signals corresponding to the mixed signals of H-a and H-b of the noninverted and inverted aromatic protons appeared.

**Figure 4:**
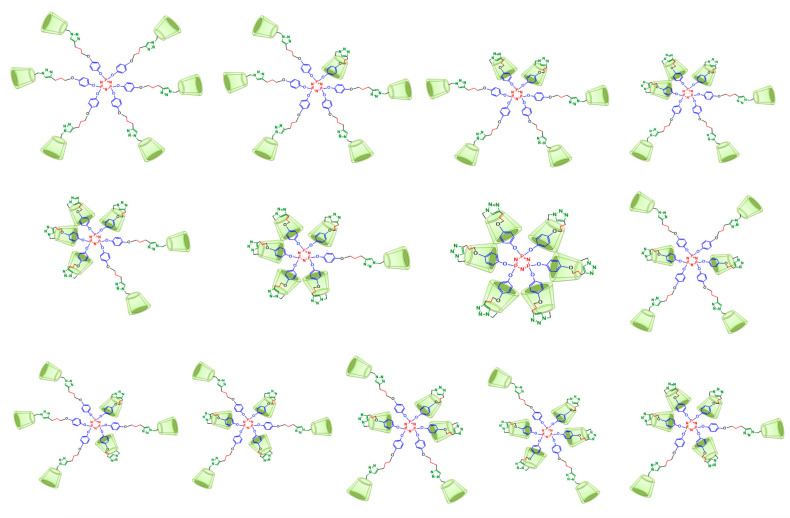
The 13 possible conformations for dendritic compound (**I**) with 0 to 6 inverted cavities. Similar conformations are expected for compound (**II**).

A detailed analysis of the groups of signals between 7.63–6.46 ppm allowed the identification of the different protons of the platforms. The H-a***i*** and H-b***i*** aromatic protons of the branches bearing a reversed CD cavity correspond to the signals between 6.92–6.89 ppm. The group of signals between 6.81–6.67 ppm is due to the H-a and H-b aromatic protons of the branches bearing a nonreversed CD and to an additional proton assigned to an inverted triazole group. The integration of the signals between 7.63–6.46 ppm indicates that 5 out of 30 integrated protons correspond to the aromatic protons of five out of six triazole groups, meaning that, on average, at least one of the CD cavities underwent a tumbling process. Nevertheless, the above-mentioned multiple signals for different triazoles demonstrate an alternating dynamic tumbling phenomenon on all CDs in the periphery of dendritic compounds (**I**) and (**II**, see Figure S3 in SI). In this way, the complexity of the signals between 7.63–7.27 ppm can be explained by the possible existence of 13 conformations for dendritic compounds (**I**) and (**II**) in water (see Figure 4).

**Figure 5:**
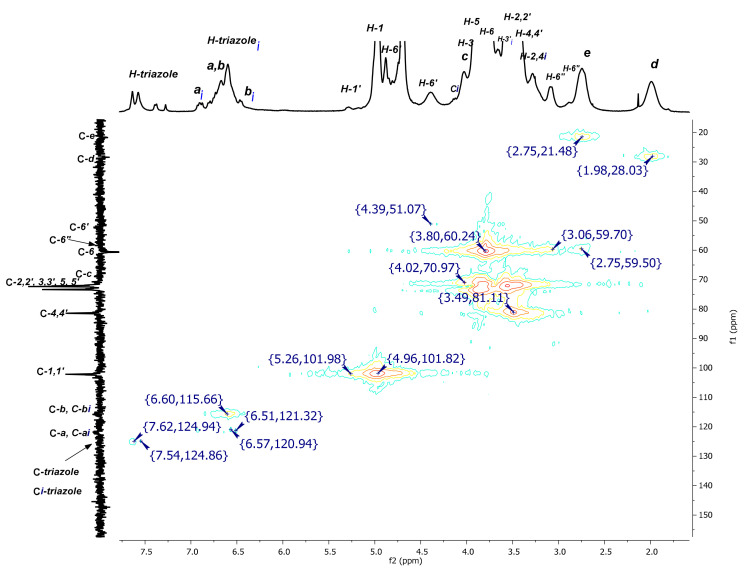
2D NMR HMQC spectrum of P_3_N_3_-[O-C_6_H_4_-O-(CH_2_)_3_-βCD]_6_ (**I**) in D_2_O.

Apart from the changes observed in the aromatic region of dendritic platforms (**I**) and (**II**) in D_2_O, other regions of the spectrum were also affected due to the tumbling effect. Unequivocal assignments of the proton signals were made based on the previous analysis performed in DMSO-*d_6_* [14], combined with the information generated from 2D NMR experiments in D_2_O. First, the 2D HMQC spectrum (see Figure 5) gave the confirmation for chemical shifts values of the multiple triazoles and inverted aromatic protons that correlated with distinctive carbon atoms in the region between 124.94–115.66 ppm of the ^13^C-NMR spectrum. Further analysis allowed the assignment of the rest of the CD proton signals and the aliphatic chain of the linker. From the down-field shift to up-field shift, it was possible to identify the H-1 and H-1′ protons of the modified and normal glucopyranose, respectively. Immediately after these signals, the nonequivalent diastereotopic protons H-6′ appeared as two sets of two signals: one set originating at 4.87 ppm and the other at 4.39 ppm, with both signals correlated to the same carbon at 51.07 ppm. It is important to notice that the tumbling of the CD cavity has the same impact on the Hc proton, as an additional signal appeared at 4.14 ppm, which correlated with the carbon signal at 70.97 ppm. This signal can only be due to one Hc***i*** because the rest of the group of signals was corresponding to the noninverted Hc protons. A similar phenomenon was observed for the signals at 3.49 ppm that correspond to H-2,2′, 4,4′ protons, which were affected by the surrounding tumbling process in the same manner. The rest of the signals were in concordance with the assignment made in DMSO-*d_6_*.

**Figure 6:**
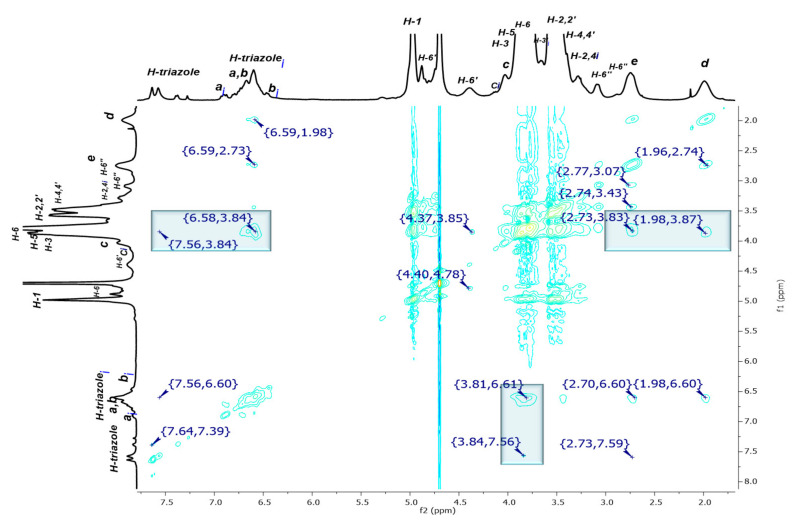
2D NMR NOESY spectrum of P_3_N_3_-[O-C_6_H_4_-O-(CH_2_)_3_-βCD]_6_ (**I**) in D_2_O.

In order to demonstrate the impact of the tumbling process on the previously described signals, NOE interactions were investigated to confirm the complete inclusion of aromatic residues inside the βCD cavity. The most significant correlations between H-triazole and H-a H-b protons (normal and inverted versions) with the H-3 and H-5 internal protons of the hydrophobic cavity of βCD are highlighted inside blue squares in Figure 6.

**Figure 7:**
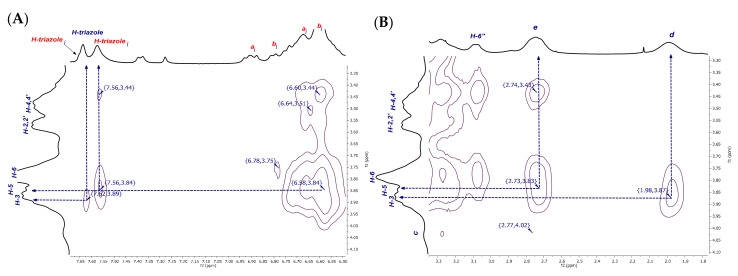
Amplification of (**A**) aromatic and (**B**) aliphatic zones in 2D NMR NOESY spectrum of P_3_N_3_-[O-C_6_H_4_-O-(CH_2_)_3_-βCD]_6_ (**I**) in D_2_O.

From a detailed examination of the 2D NMR NOESY spectrum of dendritic compound (**I**) (Figure 7A), it was observed that the H-a***i***, H-b***i***, and the H-triazole-inverted protons at 6.58 ppm and 7.56 ppm, respectively, interact with the internal signals H-3,5 at 3.84 ppm. Additional interactions are also evident, for instance, the correlations between H-a, H-b at 6.64–6.60 ppm and H-2,2′ and H-4,4′ at 3.51–3.44 ppm from CD, as well as the correlations between an inverted H-triazole proton at 7.56 ppm and the same CD signals. These observations could be explained as interactions between an inverted glucopyranose unit and a noninverted neighboring unit, as H-2,4 protons are located on the external surface of the truncated cone of the βCD. This experimental finding reinforces our proposal that the tumbling process is presented as a dynamic process. After verifying the hydrophobic interactions that account for the tumbling process, the torsion of the aliphatic chain caught our attention, as it becomes evident that H-e,d methylene protons are also interacting with the internal protons H-3,5 (see correlations between 2.73–3.83 ppm and 1.98–3.87 ppm, Figure 7B). The methylene groups undergo a strong enough torsion process to be fully included inside the βCD cavity.

### 2.2. Availability of the βCD Cavities in P_3_N_3_-[O-C_6_H_4_-O-(CH_2_)_3_-βCD]_6_ (**I**)

**Figure 8:**
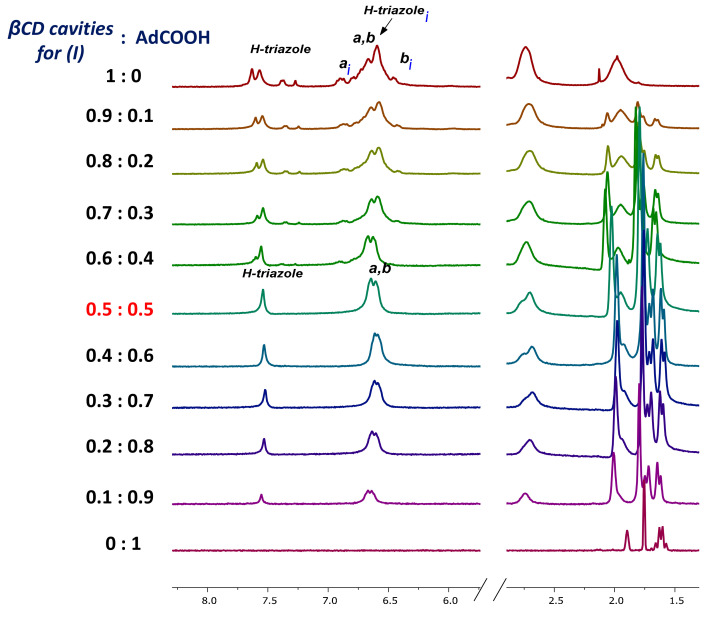
^1^H-NMR titration experiment of dendritic compound (**I**). The 5.5–8.5 ppm and 1.0–3.0 ppm regions are shown for the mixtures of dendritic compound (**I**) and AdCOOH in D_2_O with decreasing molar fraction from top to bottom, expressed with respect to the concentration in βCD cavities for dendritic compound (**I**) (XCD = [βCD]/([βCD] + [AdCOOH]). Total concentration [βCD] + [AdCOOH] = 3 mM.

After carrying out an NMR analysis of the dendritic compounds (**I**) and (**II**) without the guest, we could confirm that a dynamic tumbling process takes place in aqueous media (Scenario B, Figure 2). In this way, the tumbling process in our platforms could result in a limited capacity for inclusion complex formation, which, in principle, would block at least one of the host’s βCD cavity [17,18,39]. This is a major concern due to the potential applications in aqueous media of the presented dendrimers. In this regard, due to the high affinity of Ad for the βCD cavity [40,41,42], this molecule could be conveniently used to overcome the tumbling process of the βCD cavity. To confirm this hypothesis, we performed a titration experiment with increasing amounts of AdCOOH, recording the changes in the ^1^H-NMR spectra of platforms (**I**) and (**II**) in D_2_O (see Figure 8 and Figure S8 in SI).

**Figure 9:**
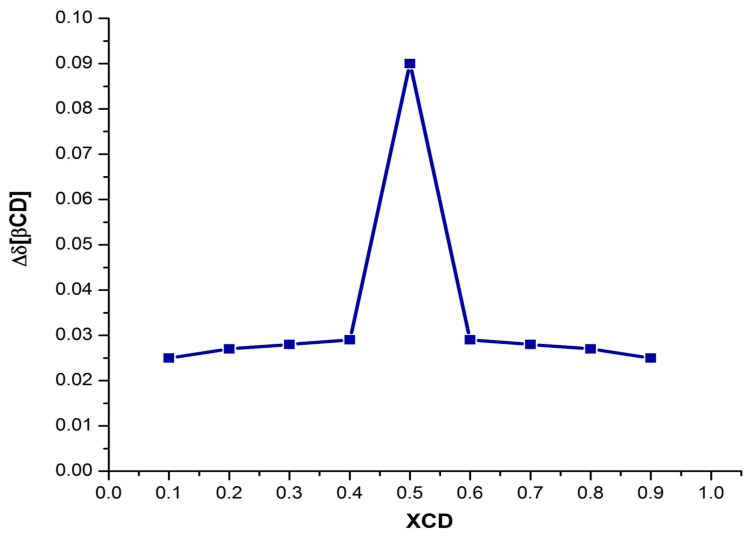
Job plot for the inclusion complex of AdCOOH with βCD cavities in the dendritic compound (**I**), [βCD] + [AdCOOH] = 3 mM at 298 K, in D_2_O. (The inflection point was found to be at 0.5, which confirms the stoichiometry 1:1.)

Each spectrum was numbered using the molar fraction XCD = [βCD]/([βCD] + [AdCOOH] for the sake of simplicity (see Figure 8 for molar ratios from 1 to 0). At a 0.9 XCD molar ratio of dendritic compound (**I**), the spectrum showed similar signals to those obtained for pure compound (**I**) in D_2_O. This behavior was maintained up to a 0.6:0.4 ratio (platform (**I**): AdCOOH). It is evident that as the XCD ratio decreased, the spectrum shape became simpler with the concomitant appearance of the “normal” multiplicity for the H-triazole and H-a, H-b protons (see region from 6 ppm to 8 ppm for the 0.5:0.5 ratio in Figure 8). Additionally, the progressive disappearance of the Hc***i*** and H-2,2′,4,4′***i*** signals reflected the reversal of the tumbling process described above. Therefore, for the 1:1 stoichiometry (see Figure 9 and Figure S9 in SI), the signal of H-triazole appeared as a singlet at 7.54 ppm, and the phenyl protons H-a H-b appeared as two doublets (*J* = 8.3, 8.1 Hz) at 6.64 ppm and 6.61 ppm, respectively. These results revealed the extended spatial conformation for dendritic compound (**I**) when forming the host–guest inclusion complex with AdCOOH, and this matches the distribution observed for pure compound (**I**) in DMSO-*d_6_* (Scenario C, Figure 2). This analysis reinforces the total disappearance of the inverted form of CD units in the periphery of dendritic compounds (**I**) and (**II**).

**Figure 10:**
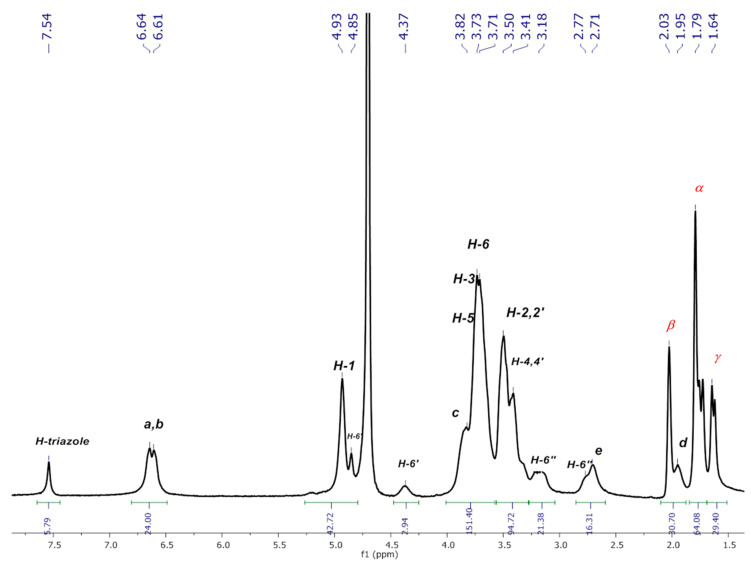
^1^H NMR spectrum of inclusion complex P_3_N_3_-[O-C_6_H_4_-O-(CH_2_)_3_-βCD]_6_ (**I**)/AdCOOH in D_2_O.

**Figure 11:**
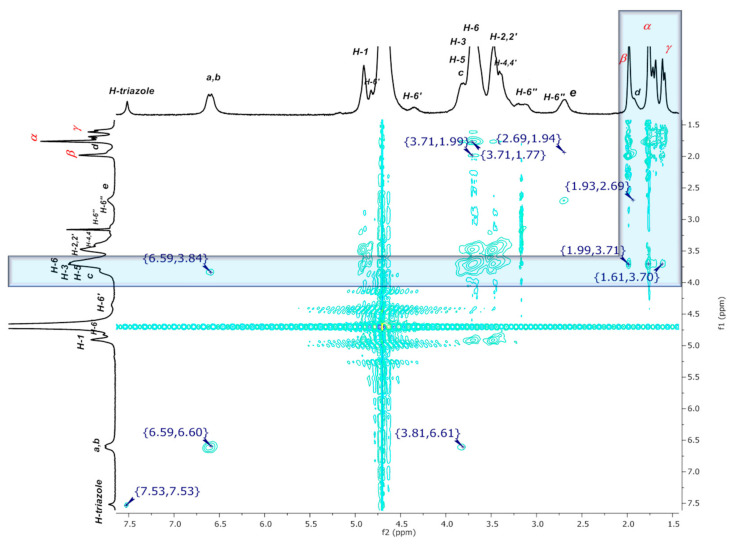
2D NMR NOESY spectrum of inclusion complex P_3_N_3_-[O-C_6_H_4_-O-(CH_2_)_3_-βCD]_6_ (**I**)/AdCOOH in D_2_O.

**Figure 12:**
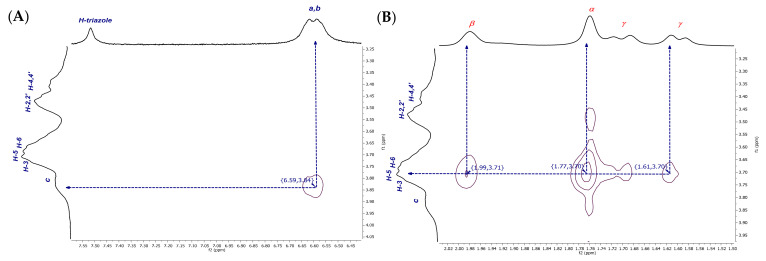
Amplification of (**A**) aromatic and (**B**) aliphatic zones in 2D NMR NOESY spectrum of inclusion complex P_3_N_3_-[O-C_6_H_4_-O-(CH_2_)_3_-βCD]_6_/AdCOOH in D_2_O.

Well-defined signals in the aromatic region can be observed in the ^1^H NMR spectrum of the inclusion complex of the dendritic compound (**I**) with AdCOOH in equimolar ratio (1:6, referred to compound (**I**):AdCOOH), as shown in Figure 10 (see Figure S10 for (**II**)). Long-range interactions were tracked through 2D NOESY NMR experiments (see Figure 11 and Figure S11 for (**II**)). Only cross-peaks between inner protons of the βCD cavity (H-3,3′; H5,5′) and those corresponding to AdCOOH (H-α; H-β; H-γ) were observed, which indicates that the CD cavities hosted only the AdCOOH molecules (see region highlighted in blue in Figure 11). Additionally, only spatial correlations between H-b/H-c were present in the NOESY spectrum of compound (**I**), and no NOE effects between triazole or aromatic protons and βCD protons were observed (see Figure 12A). Therefore, it is possible to confirm that none of the βCD cavities remained in inverted conformation. Finally, exclusive cross-peak interactions between H-α, H-β, and H-γ signals of AdCOOH and the inner protons of the CD cavity were present (see Figure 12B). The above observations confirmed the complete availability of the βCD host cavities for the AdCOOH guest, despite the initial reversed conformation in water. 

## 3. Materials and Methods

### 3.1. General Notes

All the employed reagents and solvents were purchased from Sigma Aldrich México and were used as received. The synthesis of the dendritic compounds P_3_N_3_-[O-C_6_H_4_-O-(CH_2_)_3_-βCD]_6_ and P_3_N_3_-[O-C_6_H_4_-O-(CH_2_)_4_-βCD]_6_ was carried out according to procedures previously published by our research group without modifications [14].

### 3.2. Characterization 

#### 3.2.1. NMR Experiments

Deuterated dimethyl sulfoxide (DMSO-*d_6_*) and deuterium oxide (D_2_O) with an isotopic purity of 99.9% were obtained from Cambridge Isotope Laboratories, Inc. (Cambridge, MA, USA) Tetramethylsilane (TMS), an internal NMR reference, was purchased from Sigma Aldrich Mexico. ^1^H- and ^13^C-NMR, as well as 2D HMQC, COSY, and NOESY experiments, were performed at 298 K on a Bruker Avance 400 MHz spectrometer. Chemical shifts are reported in parts per millions (ppm, δ) and coupling constants (*J*) in Hz. Multiplicities are reported using the following abbreviations: s = singlet, d = doublet, t = triplet, br = broad, and m = multiplet. The suffix letter ***i*** refers to the signals identified in an inverted conformation of the dendritic compounds.

#### 3.2.2. Characterization of P_3_N_3_-[O-C_6_H_4_-O-(CH_2_)_n_-βCD]_6_ (*n* = 3 or 4) in D_2_O

##### P_3_N_3_-[O-C_6_H_4_-O-(CH_2_)_3_-βCD]_6_

^1^H-NMR (400 MHz, D_2_O, δ ppm): 7.63–7.27 (m, 5H, H-triazole), 6.92–6.46 (m, 25H, H-a, b, H-a***i***, b***i***, H-triazole***i***), 5.29 (s, 7H, H-1′), 4.97–4.87 (m, 46H, H-1; H-6′), 4.39 (m, 7H, H-6′), 4.14–3.09(m, 302H, H-c***i***, H-c, H-5′, H-5, H-3,3′, H-6, H-2,2′, H-4,4′), 3.09 (m, 7H, H-6′′), 2.74 (m, 20H, H-6′′, H-e), 1.98 (m, 12H, H-d); ^113^C-DEPTQ NMR (101 MHz, D_2_O, δ ppm): 147.33, 145.43, 124.94, 124.86, 121.32, 120.94, 115.66, 101.82, 81.11, 70.97, 60.24, 59.70, 59.50, 51.07, 21.48, 28.03. 

##### P_3_N_3_-[O-C_6_H_4_-O-(CH_2_)_4_-βCD]_6_

^1^H-NMR (400 MHz, D_2_O, δ ppm): (7.66–7.36 (m, 5H, H-triazole), 6.84–6.14 (m, 25H, H-a, b, H-a***i***, b***i***, H-triazole***i***), 5.29 (s, 7H, H-1′), 4.98–4.79 (m, 37H, H-1; H-6′), 4.36 (m, 7H, H-6′), 4.02–3.28(m, 197H, H-c, H-5′, H-5, H-3,3′, H-6, H-2,2′, H-4,4′), 3.09 (m, 7H, H-6′′), 2.73–2.62 (m, 13H, H-6′′, H-f), 1.64 (m, 24H, H-d,e); ^13^C-DEPTQ NMR (101 MHz, D_2_O, δ ppm): 148.95, 146.05, 132.78, 127.77, 122.12, 115.28, 102.15, 81.50, 73.52, 72.39, 71.90, 60.56, 57.40, 28.52, 24.29.

#### 3.2.3. Job Plot Method 

For the Job plot method: two stock solutions, Solution Host (Sol. **H**) of P_3_N_3_-[O-C_6_H_4_-O-(CH_2_)_n_-βCD]_6_ (*n* = 3 or 4) 3 mmol/L in βCD cavities, and Solution Guest (Sol. **G**) of AdCOOH 3 mmol/L, were prepared in D_2_O. With these solutions, a series of nine samples in NMR tubes containing both P_3_N_3_-[O-C_6_H_4_-O-(CH_2_)_n_-βCD]_6_ (*n* = 3 or 4) and AdCOOH with a total concentration ([AdCOOH]+[P_3_N_3_-[O-C_6_H_4_-O-(CH_2_)_n_-βCD]_6_]) fixed at 3 mmol/L were prepared. This was accomplished by introducing increasing portions of 50 up to 500 μL of Sol. **H** in the adequate NMR tube, and then in the corresponding tube, decreasing amounts were added starting from 500 up to 50 μL of Sol. **G**. Thus, solutions with constant volume and varying βCD molar fractions (XCD = [P_3_N_3_-[O-C_6_H_4_-O-(CH_2_)_n_-βCD]_6_]/([P_3_N_3_-[O-C_6_H_4_-O-(CH_2_)_n_-βCD]_6_] + [AdCOOH])) in a complete range (0.1 < r < 0.9) were obtained. The NMR measurements were performed using the signal of D_2_O as an internal standard. The continuous variation in the ^1^H NMR chemical shift change “Δδ x XCD” (Δδ taken for the adamantyl H-γ, see Figure 9 for (**I**) and Figure S9 in SI for (**II**)) was plotted against XCD.

#### 3.2.4. Formation of Inclusion Complexes between P_3_N_3_-[O-C_6_H_4_-O-(CH_2_)_n_-βCD]_6_ (*n* = 3 or 4) and AdCOOH

The host–guest inclusion complexes between P_3_N_3_-[O-C_6_H_4_-O-(CH_2_)_n_-βCD]_6_ (*n* = 3 or 4) and AdCOOH were prepared according to the procedures previously reported [42,43]. A MeOH solution of AdCOOH was added to the aqueous solution of P_3_N_3_-[O-C_6_H_4_-O-(CH_2_)_n_-βCD]_6_ (*n* = 3 or 4) with adequate stoichiometry under vigorous stirring. After 24 h, the resulting translucent solution was filtered using a 0.45 μm membrane and lyophilized to give the corresponding supramolecular assembly. 

## 4. Conclusions

1D and 2D NMR spectroscopic studies were carried out to determine the real conformation of two dendritic compounds containing a cyclotriphosphazene core and six βCD units in the periphery in D_2_O solution. We corroborated that a dynamic tumbling process takes place at the glucopyranose unit directly linked to the spacer that binds the βCD to the cyclotriphosphazene core. This implies that a dynamic process with at least one linker chain is completely included in at least one of the six βCD cavities, resulting in a significant limitation of these dendritic compounds to form inclusion complexes. We have investigated whether this tumbling phenomenon was reversible for both dendritic compounds by studying the association to a guest molecule with high-affinity to the inner βCD cavity such as AdCOOH. A titration experiment was carried out by increasing the guest concentration. The NOE interactions shown in the NOESY spectra demonstrated that the tumbling process can be completely reversed for all βCD units in both dendritic compounds (**I**) and (**II**) with 1:1 stoichiometry. This shows that the stability of the host–guest complexes, as indicated by their association constants, is important for the reversibility of the tumbled βCD units. Finally, we demonstrated that dendritic compounds (**I**) and (**II**) are potential carriers for drugs containing a linked adamantane ring. 

## Data Availability

Data are available within this article and in the associated Supplemental Materials.

## Supplementary Materials

The following are available online at https://www.mdpi.com/article/10.3390/ph14060556/s1. Figure S1: DEPTQ-NMR spectrum of P_3_N_3_-[O-C_6_H_4_-O-(CH_2_)_3_-βCD]_6_ (**I**) in D_2_O; Figure S2: 2D NMR COSY spectrum of P_3_N_3_-[O-C_6_H_4_-O-(CH_2_)_3_-βCD]_6_ (**I**) in D_2_O; Figure S3: ^1^H NMR spectrum of P_3_N_3_-[O-C_6_H_4_-O-(CH_2_)_4_-βCD]_6_ (**II**) in D_2_O; Figure S4: DEPTQ-NMR spectrum of P_3_N_3_-[O-C_6_H_4_-O-(CH_2_)_4_-βCD]_6_ (**II**) in D_2_O; Figure S5: 2D NMR HMQC spectrum of P_3_N_3_-[O-C_6_H_4_-O-(CH_2_)_4_-βCD]_6_ (**II**) in D_2_O; Figure S6: 2D NMR NOESY spectrum of P_3_N_3_-[O-C_6_H_4_-O-(CH_2_)_4_-βCD]_6_ (**II**) in D_2_O; Figure S7: 2D NMR COSY spectrum of P_3_N_3_-[O-C_6_H_4_-O-(CH_2_)_4_-βCD]_6_ (**II**) in D_2_O; Figure S8: ^1^H-NMR titration experiment of dendritic compound (**II**); Figure S9: Job plot for the inclusion complex of AdCOOH with βCD cavities in the dendritic compound (**II**); Figure S10: ^1^H NMR spectrum of inclusion complex P_3_N_3_-[O-C_6_H_4_-O-(CH_2_)_4_-βCD]_6_ (**II**)/AdCOOH in D_2_O; Figure S11: 2D NMR NOESY spectrum of inclusion complex P_3_N_3_-[O-C_6_H_4_-O-(CH_2_)_4_-βCD]_6_ (**II**)/AdCOOH in D_2_O.
