# Supplementary material for: Guest-Mediated Reversal of the Tumbling Process in Phosphorus-Dendritic Compounds Containing β-Cyclodextrin Units: An NMR Study
Source: Pharmaceuticals (Basel). 2021 Jun 11;14(6):556. doi: 10.3390/ph14060556 (PMC8230630; doi:10.3390/ph14060556)
Supplement: Supplementary file 1 [file pharmaceuticals-14-00556-s001.zip › pharmaceuticals-1248976-SI.pdf]

Supplementary Information

Kendra Sorroza-Martínez [1], Israel González-Méndez [1,*], Mireille Vonlanthen [1], Fabián Cuétara-Guadarrama [1], Javier Illescas [2], Xiao Xia Zhu [3] and Ernesto Rivera [1,*]

Analysis data of

Figure S1. DEPTQ-NMR spectrum of P_3N_3-[O-C_6H_4-O-$(CH_2)_3$-βCD]$_6$ (**I**) in D_2O. p2

Figure S2. 2D NMR COSY spectrum of P_3N_3-[O-C_6H_4-O-$(CH_2)_3$-βCD]$_6$ (**I**) in D_2O. p3

Figure S3. ^{1}H NMR spectrum of P_3N_3-[O-C_6H_4-O-$(CH_2)_4$-βCD]$_6$ (**II**) in D_2O. p4

Figure S4. DEPTQ-NMR spectrum of P_3N_3-[O-C_6H_4-O-$(CH_2)_4$-βCD]$_6$ (**II**) in D_2O. p5

Figure S5. 2D NMR HMQC spectrum of P_3N_3-[O-C_6H_4-O-$(CH_2)_4$-βCD]$_6$ (**II**) in D_2O. p6

Figure S6. 2D NMR NOESY spectrum of P_3N_3-[O-C_6H_4-O-$(CH_2)_4$-βCD]$_6$ (**II**) in D_2O. p7

Figure S7. 2D NMR COSY spectrum of P_3N_3-[O-C_6H_4-O-$(CH_2)_4$-βCD]$_6$ (**II**) in D_2O. p8

Figure S8. ^{1}H-NMR titration experiment of dendritic compound (**II**). p9

Figure S9. Job plot for the inclusion complex of AdCOOH with βCD cavities in the dendritic compound (**II**). p10

Figure S10. ^{1}H NMR spectrum of inclusion complex P_3N_3-[O-C_6H_4-O-$(CH_2)_4$-βCD]$_6$ (**II**)/AdCOOH in D_2O. p11

Figure S11. 2D NMR NOESY spectrum of inclusion complex P_3N_3-[O-C_6H_4-O-$(CH_2)_4$-βCD]$_6$ (**II**)/AdCOOH in D_2O. p12

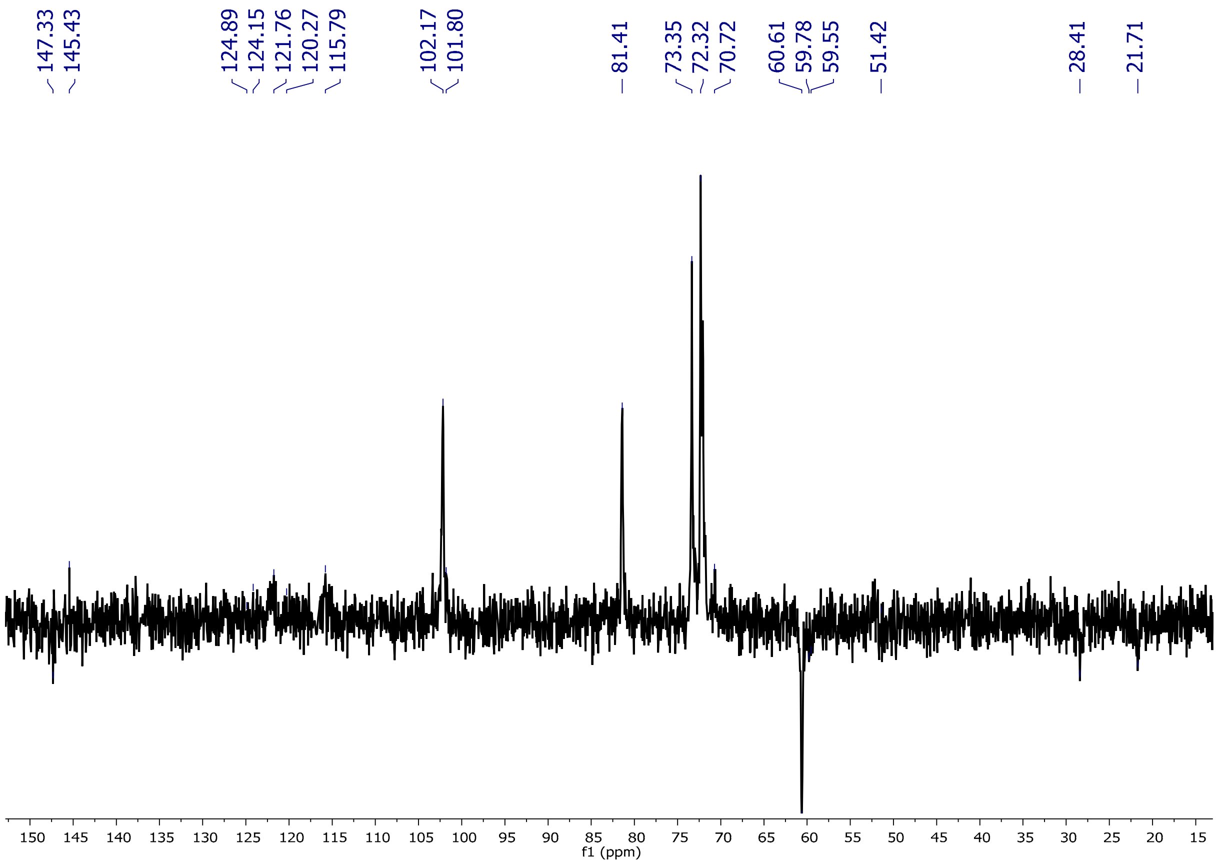

Figure S1. DEPTQ-NMR spectrum of P_3N_3-[O-C_6H_4-O-$(CH_2)_3$-βCD]$_6$ (**I**) in D_2O.

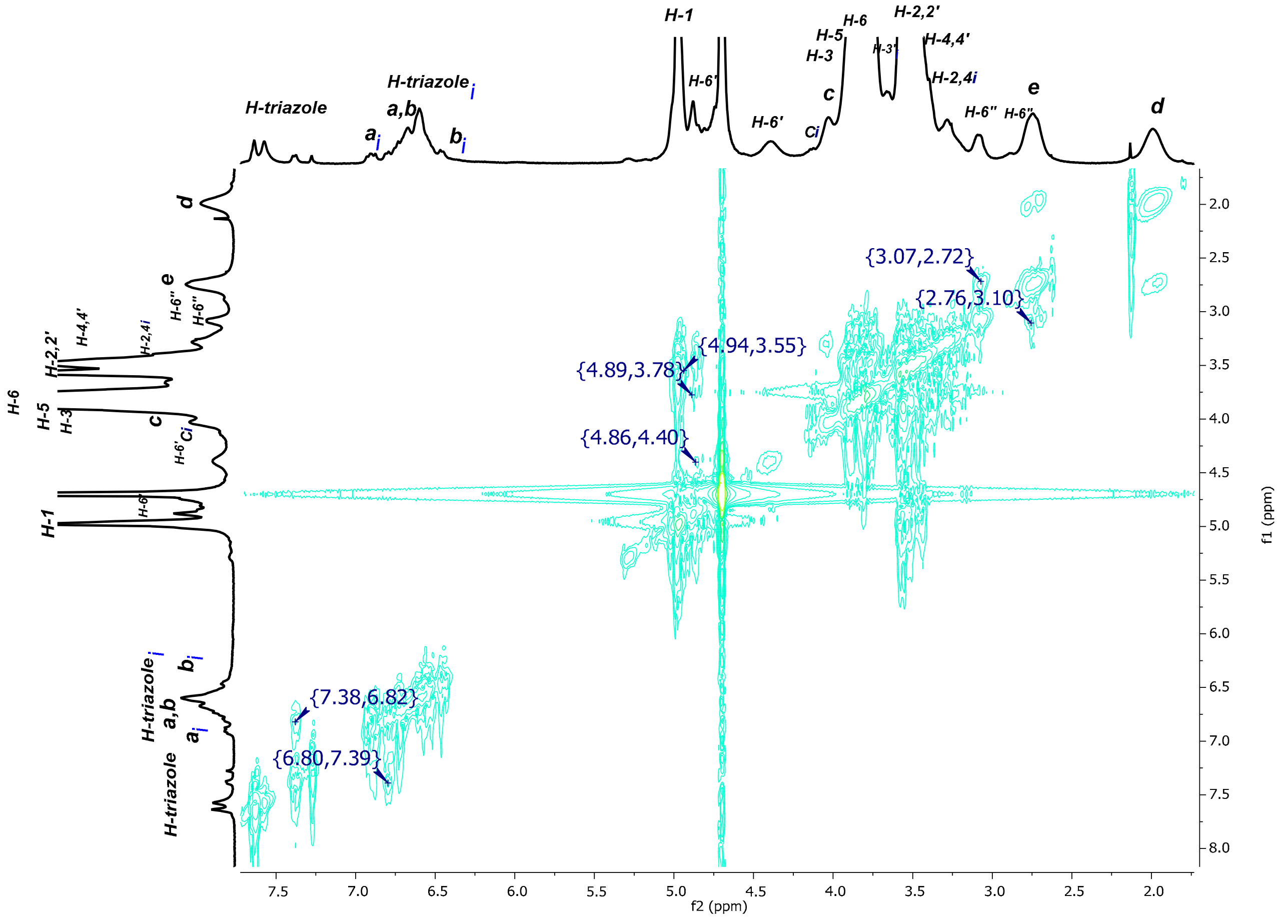

Figure S2. 2D NMR COSY spectrum of P_3N_3-[O-C_6H_4-O-$(CH_2)_3$-βCD]$_6$ (**I**) in D_2O.

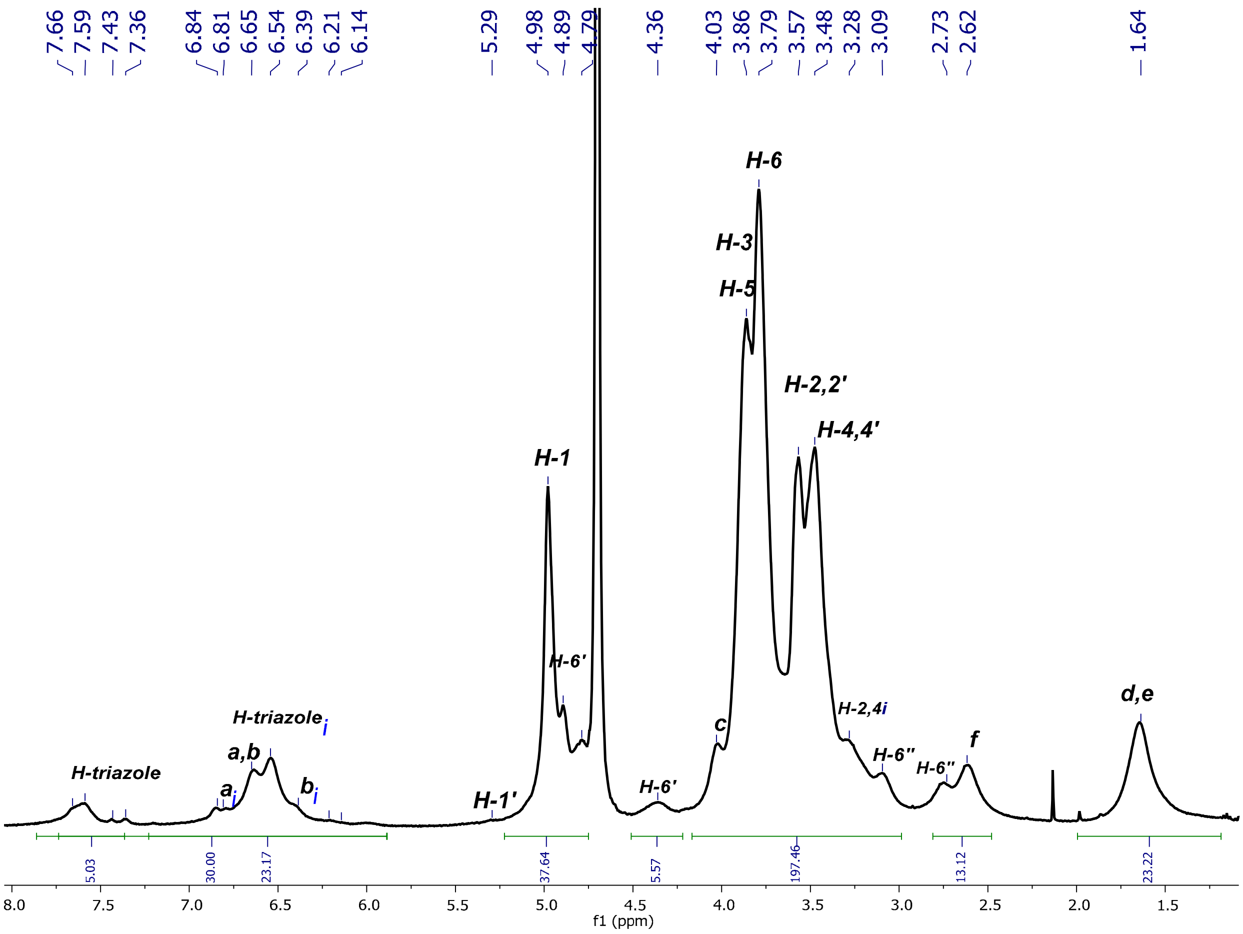

Figure S3. ^{1}H NMR spectrum of P_3N_3-[O-C_6H_4-O-$(CH_2)_4$-βCD]$_6$ (**II**) in D_2O.

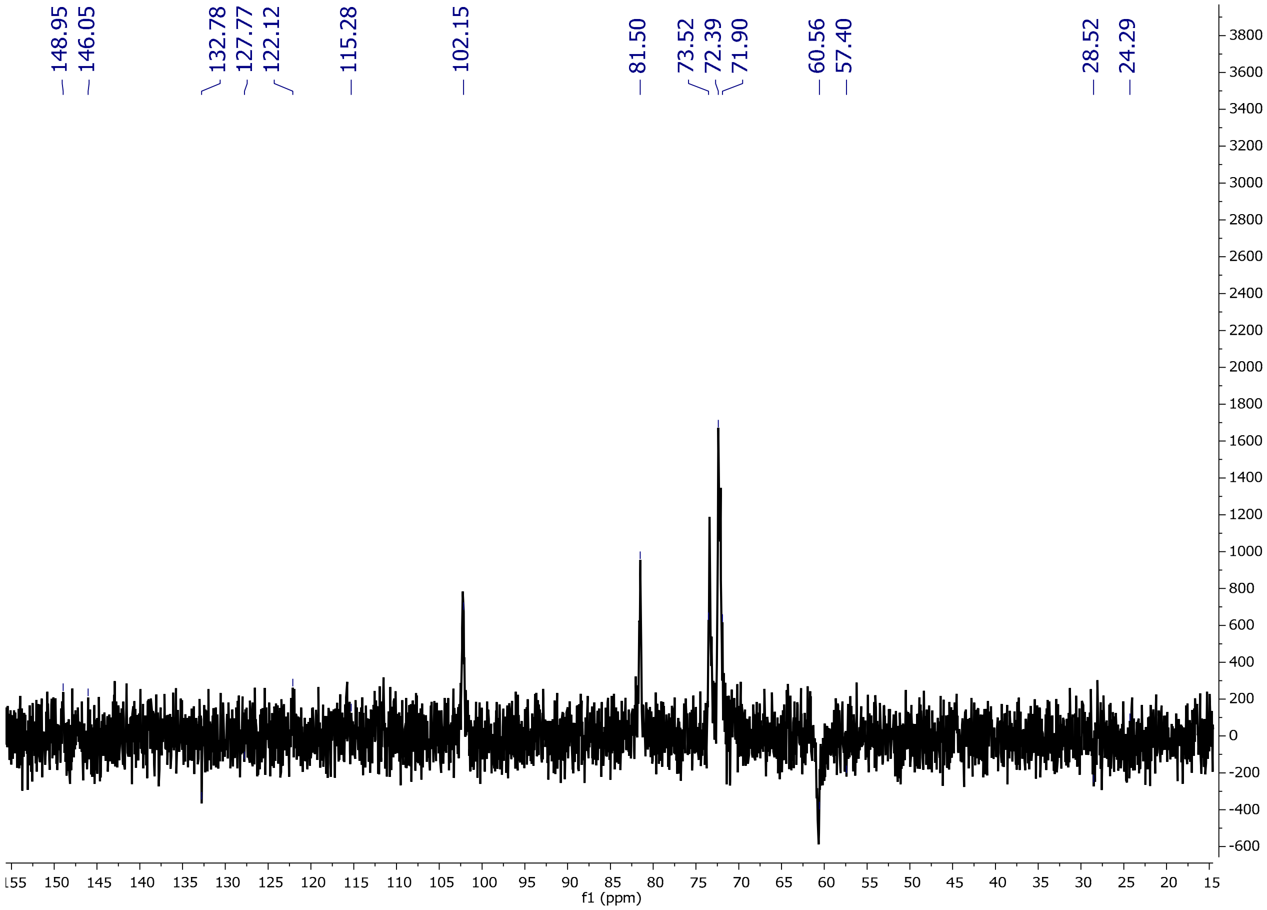

Figure S4. DEPTQ-NMR spectrum of P_3N_3-[O-C_6H_4-O-$(CH_2)_4$-βCD$]_6$ (**II**) in D_2O.

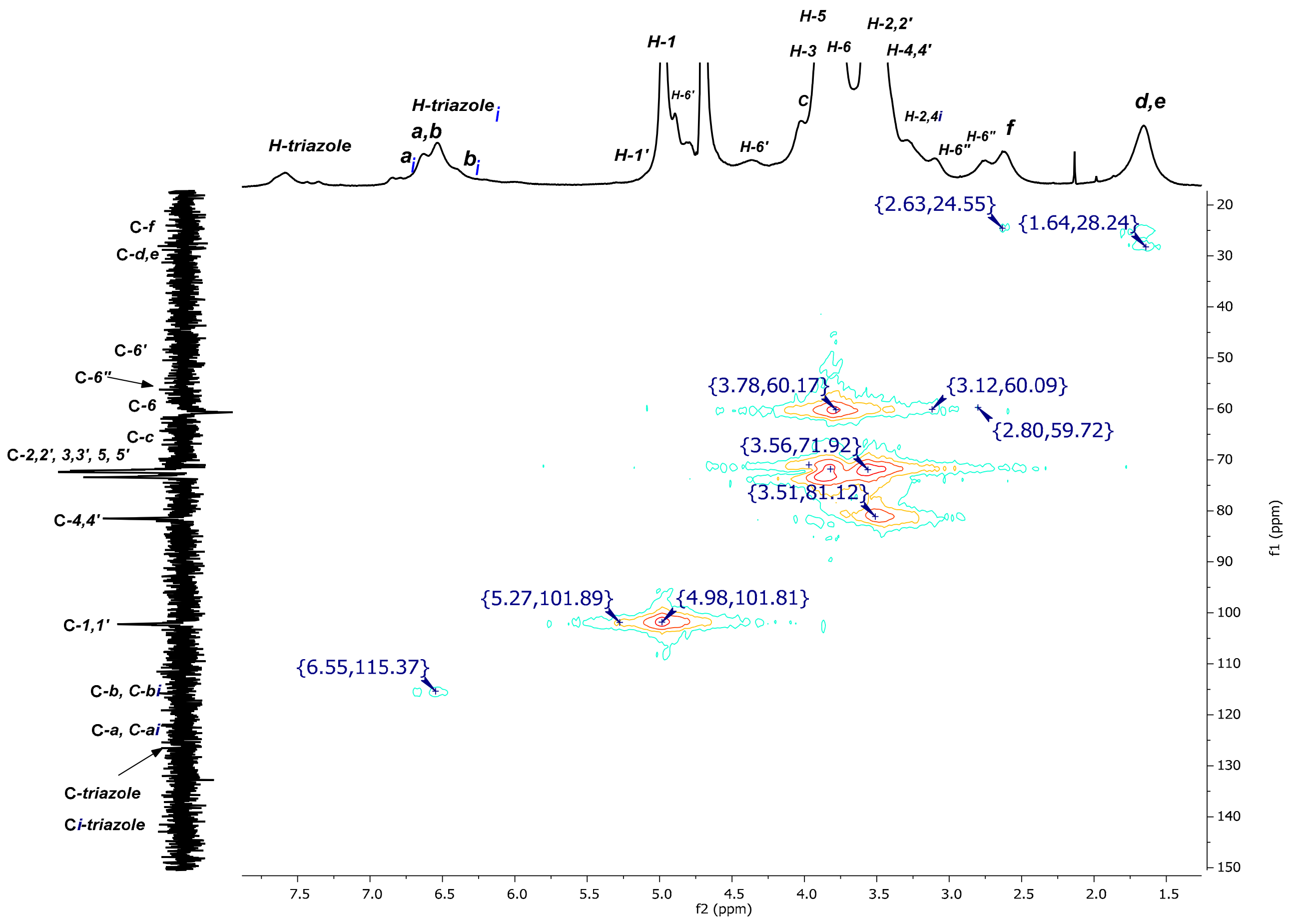

Figure S5. 2D NMR HMQC spectrum of P_3N_3-[O-C_6H_4-O-$(CH_2)_4$-βCD]$_6$ (**II**) in D_2O.

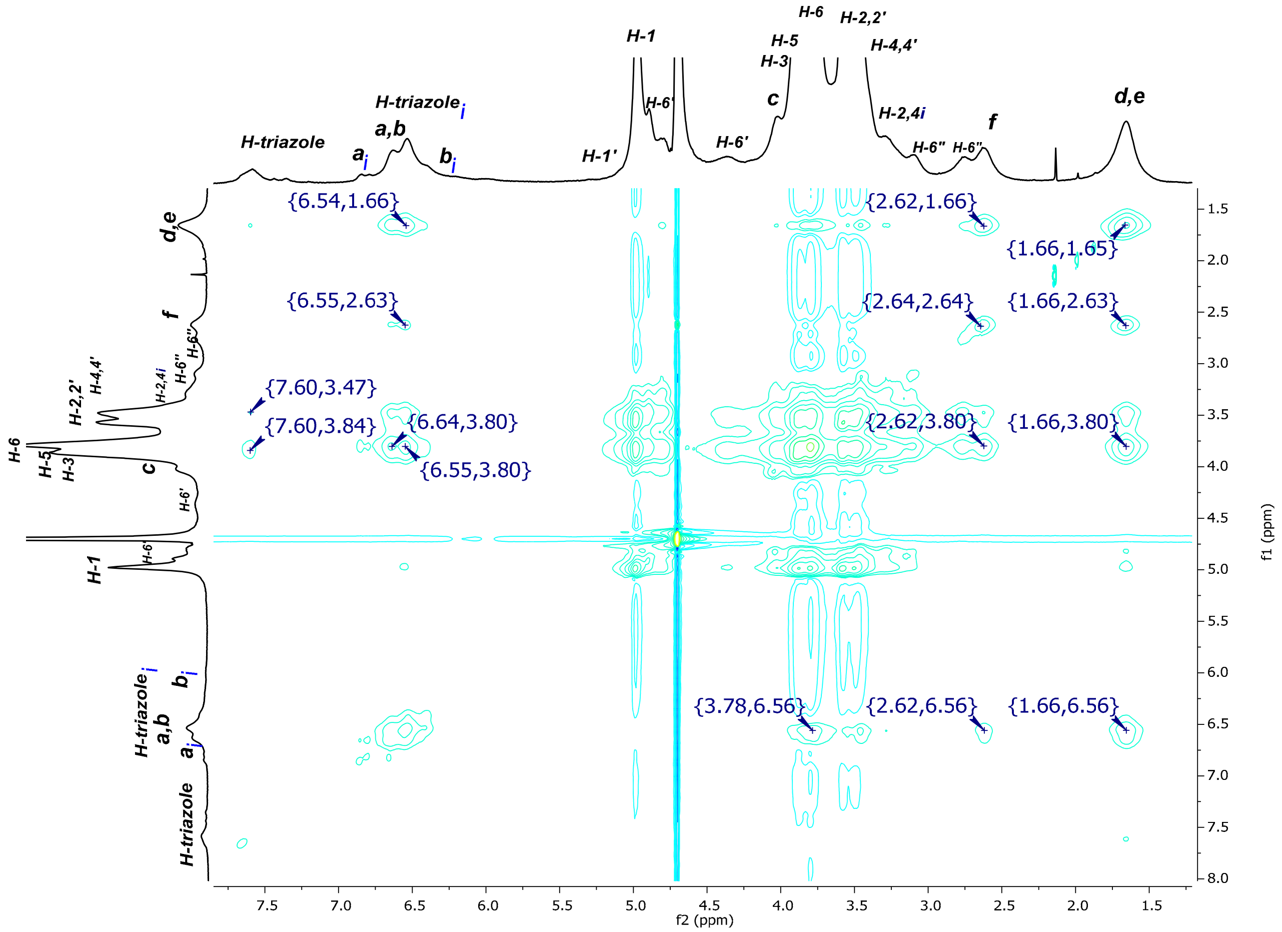

Figure S6. 2D NMR NOESY spectrum of P_3N_3-[O-C_6H_4-O-$(CH_2)_4$-βCD]$_6$ (**II**) in D_2O.

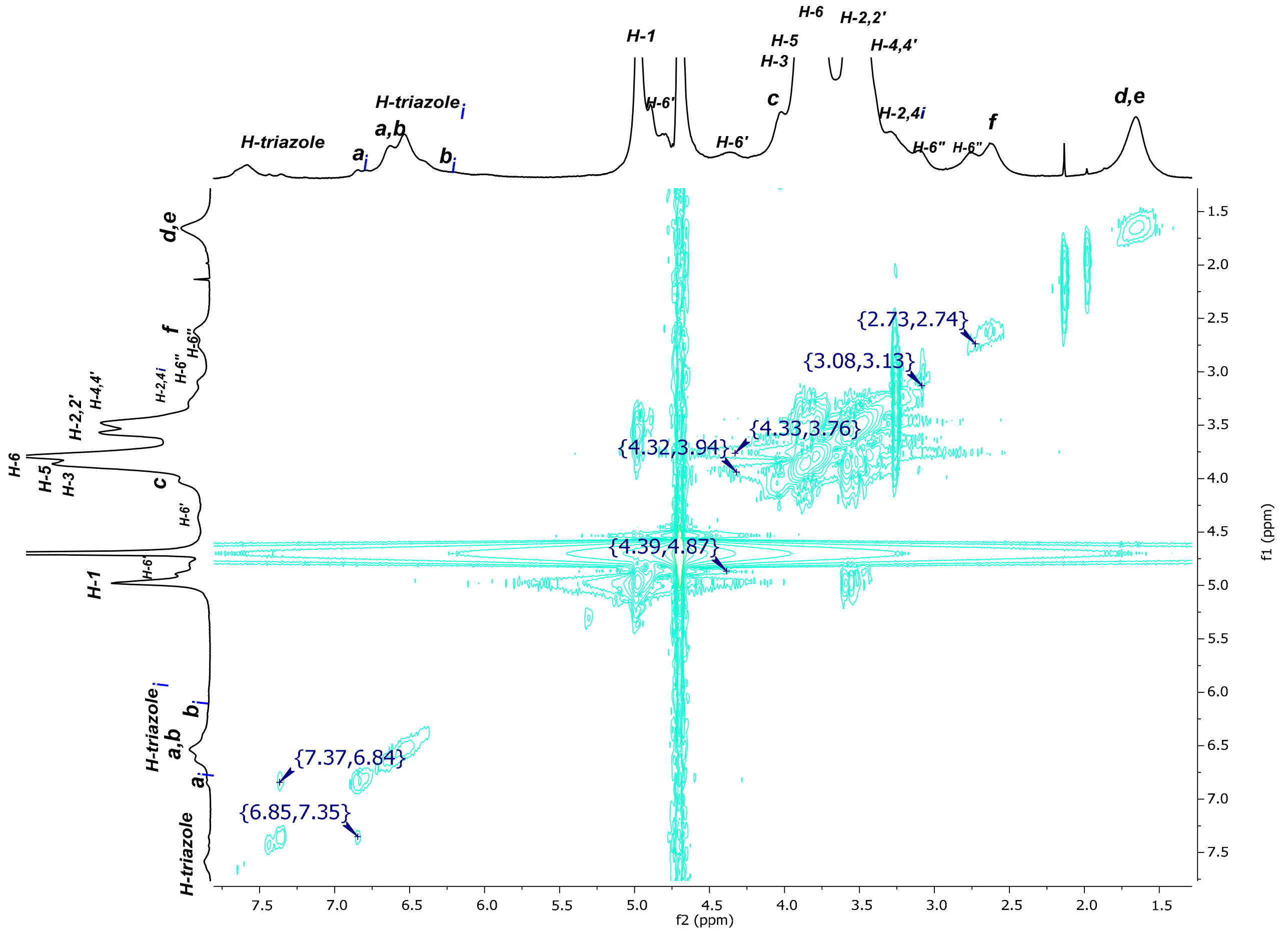

Figure S7. 2D NMR COSY spectrum of P_3N_3-[O-C_6H_4-O-$(CH_2)_4$-βCD$]_6$ (**II**) in D_2O.

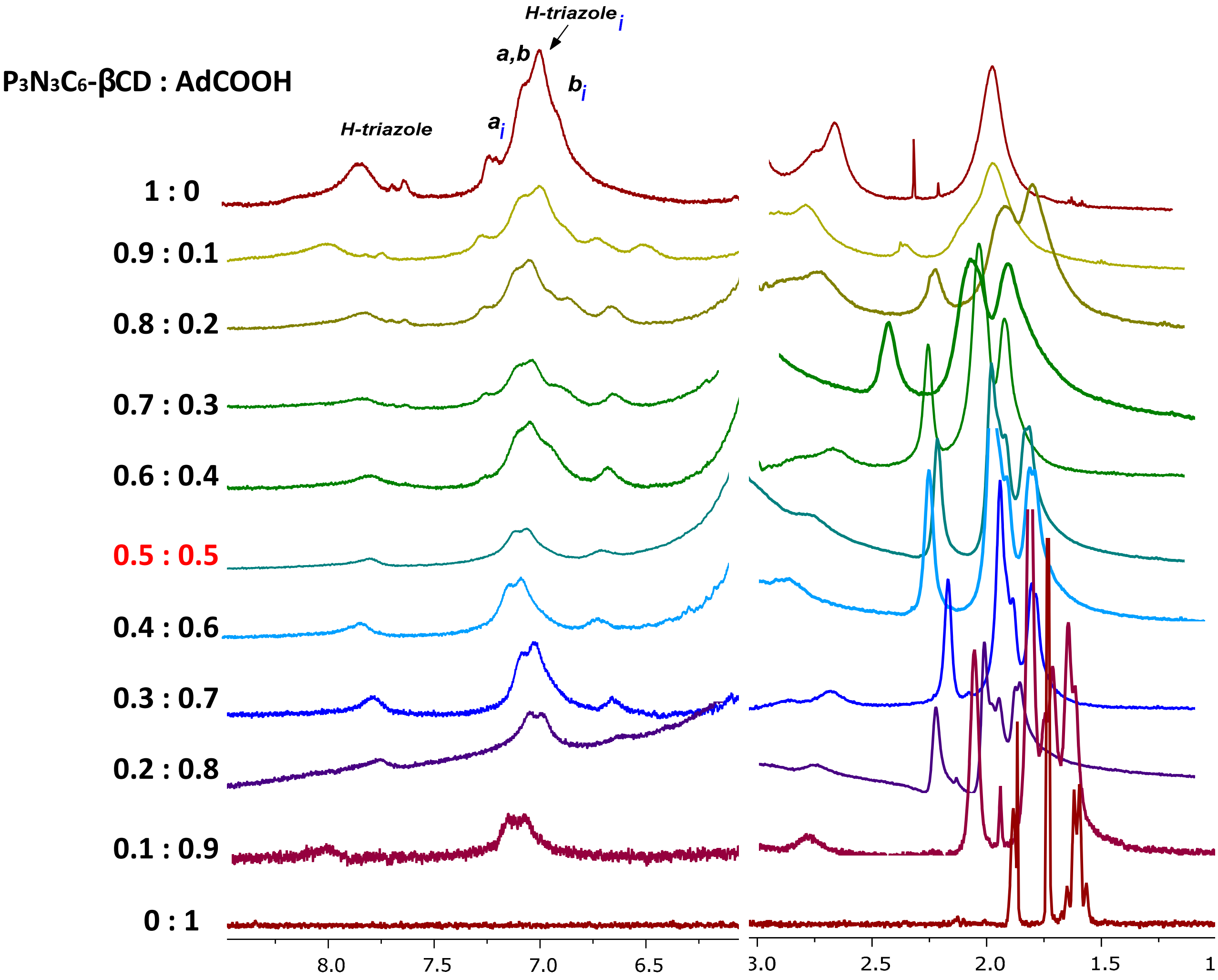

Figure S8. ^{1}H-NMR titration experiment of dendritic compound (**II**).

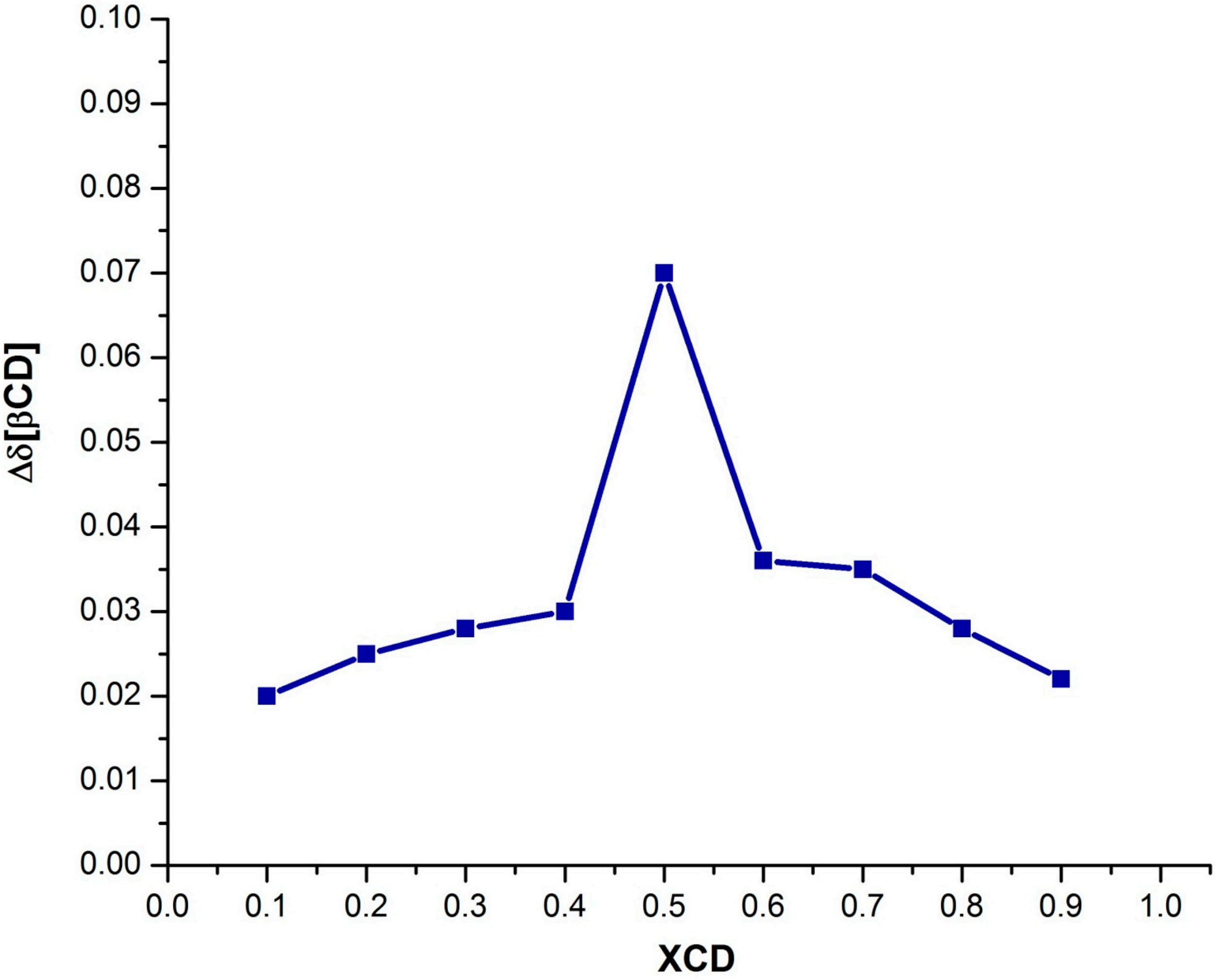

Figure S9. Job plot for the inclusion complex of AdCOOH with βCD cavities in the dendritic compound (**II**), [βCD]+[AdCOOH] = 3 mM at 298 K, in D_2O. (The inflection point was found to be at 0.5, which confirms the stoichiometry 1:1).

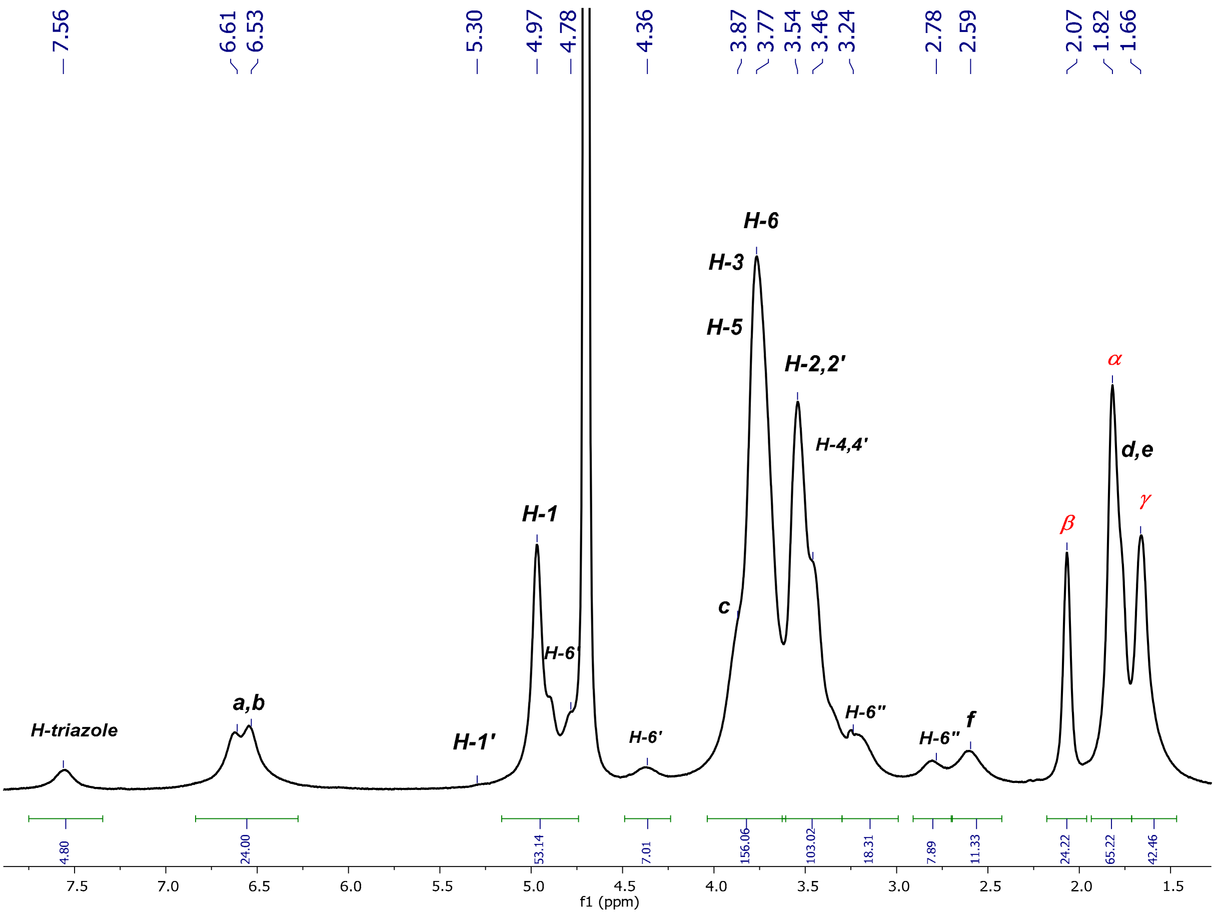

Figure S10. ^1H NMR spectrum of inclusion complex P_3N_3-[O-C_6H_4-O-$(CH_2)_4$-βCD]$_6$ (**II**)/AdCOOH in D_2O.

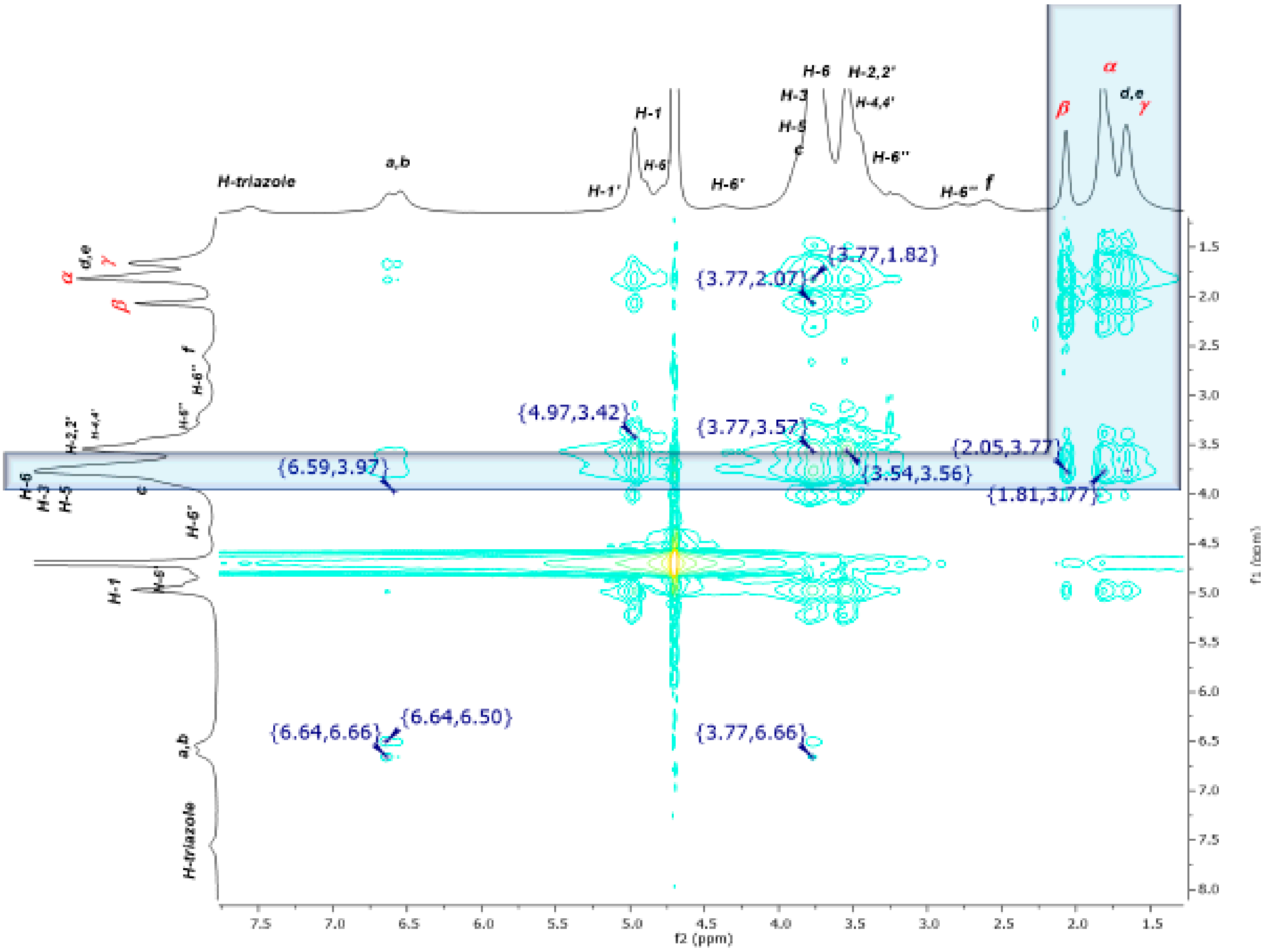

Figure S11. 2D NMR NOESY spectrum of inclusion complex P_3N_3-[O-C_6H_4-O-$(CH_2)_4$-βCD]$_6$ (**II**)/AdCOOH in D_2O.
